# Structure-based modeling of critical micelle concentration (CMC) of anionic surfactants in brine using intelligent methods

**DOI:** 10.1038/s41598-023-40466-1

**Published:** 2023-08-17

**Authors:** Danial Abooali, Reza Soleimani

**Affiliations:** 1 Young Researchers and Elite Club, Central Tehran Branch, Islamic Azad University, Tehran, Iran; 2 Department of Chemical Engineering, Faculty of Chemical Engineering, Tarbiat Modares University, P.O. Box 14115-143, Tehran, Iran

**Keywords:** Chemical engineering, Energy, Computational chemistry, Structure prediction, Chemical physics, Computational science, Crude oil

## Abstract

Critical micelle concentration (CMC) is one of the main physico-chemical properties of surface-active agents, also known as surfactants, with diverse theoretical and industrial applications. It is influenced by basic parameters such as temperature, pH, salinity, and the chemical structure of surfactants. Most studies have only estimated CMC at fixed conditions based on the surfactant’s chemical parameters. In the present study, we aimed to develop a set of novel and applicable models for estimating CMC of well-known anionic surfactants by considering both the molecular properties of surfactants and basic affecting factors such as salinity, pH, and temperature as modeling parameters. We employed the quantitative-structural property relationship technique to employ the molecular parameters of surfactant ions. We collected 488 CMC values from literature for 111 sodium-based anionic surfactants, including sulfate types, sulfonate, benzene sulfonate, sulfosuccinate, and polyoxyethylene sulfate. We computed 1410 optimized molecular descriptors for each surfactant using Dragon software to be utilized in the modelling processes. The enhanced replacement method was used for selecting the most effective descriptors for the CMC. A multivariate linear model and two non-linear models are the outputs of the present study. The non-linear models were produced using two robust machine learning approaches, stochastic gradient boosting (SGB) trees and genetic programming (GP). Statistical assessment showed highly applicable and acceptable accuracy of the newly developed models (R_SGB_^2^ = 0.999395 and R_GP_^2^ = 0.954946). The ultimate results showed the superiority and greater ability of the SGB method for making confident predictions.

## Introduction

The industrial applications of surfactant solutions demonstrate the growing importance of these systems in everyday life^1^. Surfactants are utilized in various industries, including enhanced oil recovery (EOR)^2^, cleaners and detergents^3,4^, emulsifiers and dispersing agents^5^, foods^6^, coatings^7^, and many other chemical, petroleum, and pharmaceutical processes^1^.

Surfactants are amphiphilic compounds consisting of hydrophilic (polar head) and hydrophobic (nonpolar tail) parts. Due to this unique structure, surfactants tend to accumulate at the surface of solutions such as water or brine. Once the surface is saturated with surfactant molecules, the remaining particles accumulate in the bulk and form micelles^8^.

Among different types of surfactants, anionic surfactants are known for their high foaming properties, and some industries such as chemical EOR (CEOR), detergents, and cleaners, often use them in specific applications. In the present study, we investigated several anionic surfactants to better understand their behavior and properties.

Critical micelle concentration (CMC) is an important property of surfactants that has been investigated in many theoretical and experimental studies. The CMC is defined as the maximum concentration of a surfactant at which micelles do not form or the concentration at which micelles begin to form^8,9^.

In concentrations larger than CMC, the solution is considered micellar and exhibits different behavior from a dilute solution (e.g., a solution with concentration less than the CMC). From an industrial and economic point of view, operating surfactant systems at the CMC often results in specific efficiencies. In addition, several theoretical and thermodynamic studies have been carried out to estimate various properties of surfactant systems based on the same properties at the CMC. A good example in this area is the estimation of the surface tension of a surfactant solution from the surface excess concentration at the CMC^8,9^. The CMC is a straightforward way to assess the behavior of surfactant solutes on surfaces and colloids, making it a valuable tool for evaluating their potential industrial and pharmaceutical applications^10,11^. In certain situations, it is desirable for surfactants to have a low CMC, such as when they are used to dissolve hydrophobic drugs in micellar cores with minimal surfactant quantities^10,12^. Additionally, in applications like foaming, wetting, and hard surface cleaning, where a low product surface tension is often desired, micelles act as surfactant reservoirs above the CMC, allowing for product dilution without significant changes in surface tension. On the other hand, in cases like membrane protein extraction, a high CMC is preferred since the extraction efficiency typically plateaus at around four times the CMC of the surfactant due to self-association^10,13^.

Due to the numerous applications of CMC, knowledge about the values of this specific property is essential under different conditions. Experimental measurements are a reliable way to access to accurate values. However, conducting experiments in laboratories is not always simple, especially at high temperatures and pressures. In some cases, experimental measurements are expensive and/or time-consuming and may involve uncertainties about impurities, possible decompositions, etc. The application of estimation methods and mathematical models may be effective in this area. Empirical modeling, as a famous method, and different mathematical-statistical algorithms are available for developing computational correlations. Well-known tools such as genetic programing (GP), artificial neural networks (ANNs), particle swarm optimization (PSO), adaptive neuro-fuzzy inference system (ANFIS), support vector machines (SVMs), stochastic gradient boosting (SGB) trees, etc., are applied.

In order to estimate the properties of chemical compounds, molecular based approaches such as group-contribution and quantitative structure–property relationship (QSPR) are preferred^14^. In the group-contribution method, properties of chemical compounds are estimated by analyzing different parts of their molecular structures, such as functional groups, singular and multiple bonds, etc. This is an interesting method that can sometimes achieve high accuracy. However, there are some disadvantages, such as its limited applicability to certain isomers as well as chemical compounds with novel structure.

QSPR is another estimation approach in which the considered property (objective function) is estimated from a number of chemical parameters of the components called ”molecular descriptors”^15^. The molecular descriptors relate solely to the molecular structures of components and are calculated by applying certain mathematical rules. One of the important advantages of a QSPR model is the ability to estimate the properties of newly designed chemical compounds only solely from their molecular descriptors. In this study, the QSPR technique was applied to produce novel models for CMC as functions of molecular descriptors.

There are several mathematical models for estimating the CMC of anionic surfactants. In 1953, Klevens^16^ proposed a relationship between the CMC and the number of carbon atoms in the surfactant tail (N) as follows:

1 $$\log \, ({\text{CMC}}) \, = {\text{ A}} - {\text{BN}}$$

A and B are constants for homologue series of surfactants under fixed condition. This model is simple, but it is valid for fixed conditions and structurally simple surfactants.

In the main studies of CMC modelling, the QSPR approach has been used. Huibers et al.^17^ developed a multi-variable linear model based on QSPR from a data set of 119 anionic surfactants at 40 °C. The model is as follows:

2 $$\begin{aligned} \log_{ \, 10} ({\text{CMC}}) \, & = \, (1.89 \, \pm \, 0.11) - (0.314 \, \pm \, 0.01){\text{ t - sum - KH}}0 \, - \, (0.034 \, \pm \, 0.003){\text{TDIP}} \\ & \quad - (1.45 \, \pm \, 0.18){\text{ h - sum - RNC}} \\ \end{aligned}$$

In this equation, the descriptor “t-sum-KH0”, which is the zeroth-order Kier and Hall molecular connectivity index, is considered as a variable for the hydrophobic part (tail) of the surfactant. This parameter is related to the molecular volume and surface area. “TDIP” represents the total dipole moment of the surfactant and is a descriptor for the entire molecule.“h-sum-RNC” is the relative number of carbon atoms in the hydrophilic moiety (head) and reflects the diversity of head group structures^18^.

Huibers et al.^17^ also developed a multi-variable linear correlation for the types of sulfate and sulfonates using 66 data points at 40 °C:

3 $$\begin{aligned} \log_{ \, 10} ({\text{CMC}}) \, & = \, (2.42 \pm 0.07) - (0.537 \, \pm \, 0.009){\text{ KH}}1 - (0.019 \, \pm \, 0.002){\text{ KS}}3 \, \\ & \quad + \, (0.096 \, \pm \, 0.005){\text{ HGP}} \\ \end{aligned}$$

KH1 is the first-order Kier and Hall molecular connectivity index, which is a parameter that correlates with molecular volume and surface area. KS3 is the of third-order Kier shape index that is related to molecular shape. HGP determines the carbon number attached to the hydrophilic moiety and is located on the longest chain of the surfactant's molecule^17,18^.

Another linear model was produced by Jalali-Heravi and Konouz^19^ using 31 anionic surfactants (27 alkyl sulfates and 4 alkane sulfonates) at 40 °C. The correlation was presented as follows:

4 $$\begin{aligned} \log_{ \, 10} ({\text{CMC}}) \, & = - \, (3.1373 \, \pm \, 0.4374) - (9.7401 \, \pm \, 1.3165) \times 10^{ - 4} \times {\text{WI }} \\ & \quad + \, (11.0284 \, \pm \, 2.2709){\text{RA}}^{ - 1} \, + \, (6.704 \, \pm \, 0.6150){\text{ D}} \\ \end{aligned}$$

In this equation, WI, which is the Wiener number, a topological descriptor that measures molecule compactness. RA^−1^ is the reciprocal of Randic index, a criterion for quantifying molecular branching and D is the molecular dipole moment.

In 2002, Wang et al.^20^ proposed a QSPR linear model for 40 anionic surfactants. This model involved a number of quantum mechanical descriptors:

5 $$\begin{aligned} \log_{ \, 10} ({\text{CMC}}) & = 0.546 - 0.269{\text{KH}}0 - 0.0037 \, \Delta {\text{H}}_{{\text{f}}} + \, 0.000224{\text{ E}}_{{\text{t}}} + \, 0.382{\text{ E}}_{{{\text{HOMO}}}} \\ & \quad + 0.493{\text{ E}}_{{{\text{LUMO}}}} - 0.0134{\text{ D}} \\ \end{aligned}$$

In this equation, KH0, E_t_, ΔH_f_, E_HOMO_ and E_LUMO_ represent the Kier and Hall molecular connectivity index of zeroth order, total energy of the molecule, molar heat of formation, energy of the highest occupied molecular orbital, and energy of the lowest unoccupied molecular orbital, respectively.

The model of Robert et al.^21^ was another correlation produced in 2002 which was generated by adopting the octanol/water partition coefficient for 16 anionic surfactants, including primary alcohol sulfate and primary alcohol ester sulfate at 50 °C. They applied two variables in their correlation: Π_h_, which is the octanol/water partition coefficient of the hydrophobic moiety and is defined as the octanol/water partition coefficient of the whole molecule minus the octanol/water partition coefficient of the negatively charged fragment SO_3_^−^ or OSO_3_^−^^18^, and L,which is the length of hydrophobic moiety as a C–C single bond unit. The following model is their suggested correlation:

6 $${\text{log}}_{{ 10}} {\text{(CMC) = 1}}{.5 (} \pm {0}{\text{.3)}} - {0}{\text{.39 (}} \pm {0}{\text{.05) }}\Pi_{H} - {0}{\text{.08 (}} \pm {0}{\text{.02) L}}$$

A multi-variate linear model was presented by Li et al.^22^ in 2004. They optimized the hydrophobic–hydrophilic structures of 98 anionic surfactants, including sodium alkyl sulfates, sodium alkyl sulfonates, sodium alkyl benzene sulfonates, and potassium alkyl carboxylates, and calculated quantum chemical data to develop their correlation:

7 $$\begin{aligned} \log_{ \, 10} (CMC) \, & = \, (1.89 \, \pm \, 0.0671) - \, (0.0697 \, \pm \, 0.00151){\text{ N}}_{{\text{T}}} - (0.0323 \, \pm \, 0.0015){\text{D}} \\ & \quad + \, (0.381 \, \pm \, 0.0305) \, Q_{{\text{C - max}}} \\ \end{aligned}$$

In this equation, N_T_ represents the total number of atoms, and Q_C-max_ represents the maximum net atomic charges on the carbon atom.

Li et al.^23^ also developed a linear model in 2006 for 36 sodium alkyl benzene sulfonates using the same method as their previous work:

8 $$\log_{ \, 10} ({\text{CMC}}) \, = - \, 0. \, 213 - \, 0. \, 261\left\{ {{\text{KH}}0} \right\} \, + \, 0. \, 598\left\{ {{\text{f}} - {\text{I}}_{{{\text{BAL}}}} } \right\} - 0. \, 0191\left\{ {\text{D}} \right\}$$

f−I_BAL_ is the Balaban distance connectivity index of the hydrophobic segment, which stands for molecular size and compactness.

Katritzky et al.^18,24^ recommended using topological, solvation, and charge-related molecular descriptors for developing models, due to the significant driving force of the intermolecular interactions between anionic surfactants and water. However, different categories of descriptors have been used in modeling, and acceptable results have been presented.

A general investigation shows that almost all suggested mathematical correlations for estimating CMC have been constructed based on chemical descriptors in constant conditions of temperature (T), mostly in aqueous solutions without salinity. However, CMC is a physico-chemical quantity of surfactants that is highly influenced by some basic parameters. Along with the chemical structure of a surfactant, the salinity of solution, temperature (T), pressure (P), and pH are the most effective parameters on CMC, as shown in previous studies^25–29^.

The impact of temperature on the CMC of surfactants in water is intricate and follows a non-linear trend. Initially, the CMC decreases with temperature until it reaches a minimum, after which it starts to increase with a further increase in temperature. This is due to the fact that higher temperatures lead to reduced hydration of the hydrophilic part of the surfactant molecule, which facilitates the formation of micelles. However, at the same time, the increase in temperature also interferes with the structured water molecules surrounding the hydrophobic part of the surfactant molecule, which impedes micelle formation. Thus, the balance between the favorable and unfavorable effects of temperature on micellization determines whether the CMC increases or decreases over a certain temperature range^30^. Generally, the addition of salt to anionic surfactant solutions results in a reduction of surface tension, with the effect becoming more significant at higher salt concentrations. This phenomenon is attributed to the electrostatic interactions that facilitate the migration of surfactant monomers towards the interface^31^.

The amin objective of this study was to generate novel and accurate models that incorporates both the effective parameters on CMC, including chemical descriptors and physical variables, for several widely-used common anionic surfactants. In this study, the QSPR method was coupled with two robust machine-learning approaches,- SGB and GP. New predictive methods were developed with applicability and confidence for estimating CMC. of the inclusion of physical properties such as T, pH and salinity along with the chemical descriptorsfor estimating of CMC is a novel and innovative approach. Additionally, the use of SGB and GP methods to develop CMC models is a new technique.

## Materials and methods

### Data set

The total dataset includes 488 sets (i.e. observations) of experimental data adopted from the literature^11,19,25,32–42^. Each set (observation) contains basic parameters, including the salinity of the solution (in the the form of NaCl equivalent salinity), temperature (T), pH, and CMC at atmospheric pressure. The collected data involve 111 widely-used sodium-based anionic surfactants, including sodium alkyl sulfates, sodium alkane sulfonates, sodium alkyl benzene-sulfonates, sodium di-alkyl sulfosuccinate, and sodium alkyl (X) oxy-ethylene sulfates (X represents mono, di, tri or tetra).

It should be noted that NaCl equivalent salinity (S_eq_) is defined as the salinity of brine in which all dissolved salts (cations and anions) have been replaced with a certain amount of sodium chloride so that the brine resistivity keeps the same^43,44^. It is a usual and simple method for representing salinity where a common criterion (the amount of NaCl) is applied instead of a diverse variety of salts. Additionally, the pH of solutions collected in the dataset is attributed to the dissolved salts (i.e. effects of cations and anions of the salts) without the effects of surfactant ions, and there are no acid or base additives in the collected data. The ranges of all variables have been shown in Table 1.

**Table 1:** The ranges of basic variables in the present study.

Parameters		Range
Temperature	T (K)	273.15–363.15
NaCl equivalent salinity	S_eq_ (ppm)	0–70,131.36
pH	pH	6.146–11.133
Critical micelle concentration	Log _10_ (CMC)	−1.39794 to 2.99564

To generate the data-based models, the entire dataset was first randomly divided into two subsets. According to the literature^45–49^, 90% of the data was considered as training data, and the remaining data points were utilized as test data. The training dataset was used to develop the CMC model, while the test data was used to test the estimation ability of the newly developed model.

### Molecular descriptors generation

Molecular descriptors of a compound are numerical chemical specifications calculated from the chemical structure of the component. They are computed using certain mathematical rules that are available in specialized software^50,51^. Firstly, the chemical structure of the compound should be accurately drawn in an appropriate software. In the present study, the structures of surfactant ions (anions) were drawn in ChemBio3D Ultra, which is a module of the ChemBioOffice software^52^. Then, the drawn structures were optimized by minimizing the energy level using molecular mechanics (MM2). The optimized structures were saved as SDF files^53^ and fed to the Dragon software for calculating the descriptors. The online version of Dragon software is freely available^54^. Dragon software calculates different categories of descriptors, including (1) 0D-constitutional descriptors (atom and group counts), (2) 1D-functional groups and atom-centered fragments, (3) topological, autocorrelations, connectivity indices, information indices, and eigenvalue-based indices, (4) weighted holistic invariant molecular (WHIM) and geometry, topology, and atom-weights assembly (GETAWAY) descriptors, and so on. For more information about molecular descriptors, please refer to the literature^55^.

In the next step, descriptors with the same value for all compounds in the dataset, i.e.,non-informative descriptors, were excluded. Finally, a set of 1410 optimized descriptors were considered for each compound in the modeling process.

### Selection of the most informative descriptors as surfactants variables

In the QSPR approach, after computing the descriptors, a small subset of the most effective descriptors should be selected as model chemical (e.g., structural) parameters along with other (basic) variables. In other words, a small number of descriptors should be chosen from the large pool. There are different methods for subset variable selection, such as genetic algorithm-based multivariate linear regression (GA-MLR)^15^, genetic function approximation (GFA)^51^, forward stepwise regression (FSR), replacement method (RM)^56,57^, enhanced replacement method (ERM)^56,58^, and so on.

In this study, the ERM was used to select the best subset. A detailed explanation of the ERM procedure can be found elsewhere^56,58,59^. In the ERM method, the user determines the number of descriptors that the algorithm should find, and ERM will find them in the form of a multivariate linear regression. The main challenge is to determine a simple regression with a minimum number of descriptors that provides appropriate accuracy. To select the best descriptors in this study, we first attempted to find two descriptors using the training dataset. The ERM algorithm developed the best linear regression with two descriptors. Then, the number of descriptors was increased one by one to enhance the accuracy of the multivariate regression. For each regression, the correlation coefficient (R^2^) and residual standard deviation (RSD) were calculated using the following formulas:

9 $${\text{R}}^{2} \, = \, 1 \, - \, \frac{{\sum\nolimits_{i = 1}^{n} {\left( {{\text{y}}_{{\text{i}}}^{{{\text{exp}}.}} - {\text{y}}_{{\text{i}}}^{{{\text{cal}}.}} } \right)^{2} } }}{{\sum\nolimits_{i = 1}^{n} {\left( {{\text{y}}_{i}^{\exp .} - \overline{{\text{y}}}^{\exp .} } \right)^{2} } }}$$

10 $${\text{RSD}} = \sqrt {\frac{{\sum\limits_{{{\text{i}} = 1}}^{{\text{n}}} {({\text{y}}_{{\text{i}}}^{\exp .} - y_{{\text{i}}}^{{{\text{cal}}.}} )^{2} } }}{{{\text{n}} - {\text{d}} - 1}}}$$

**Figure 1:**
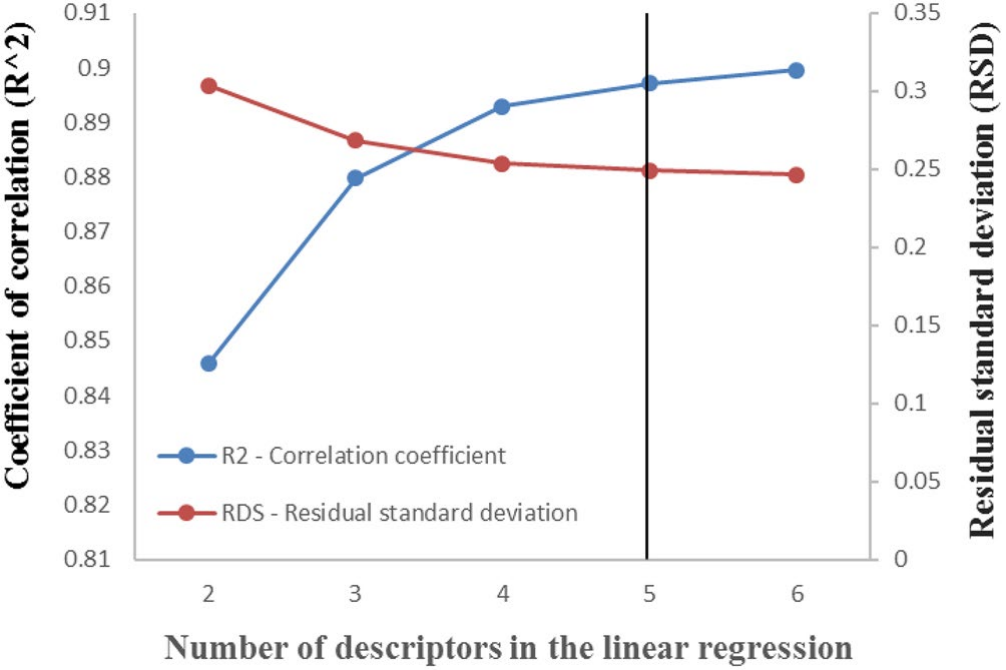
The effect of number of molecular descriptors on the prediction capability in descriptors selection step.

In the equations, ${\text{y}}_{{\text{i}}}^{{{\text{exp}}{.}}}$,${\text{y}}_{{\text{i}}}^{{{\text{cal}}{.}}}$, and $\overline{{\text{y}}}^{\exp .}$ represent the experimental, estimated, and average of experimental values of objective function (log _10_ CMC), respectively. *n* is the number of samples in the dataset (training dataset), and *d* is the number of descriptors in the linear regression. A lower value of RSD and a higher value of R^2^ are desired. The results of the descriptor selection step have been shown in Fig. 1. It can be inferred from Fig. 1 that increasing the number of descriptors beyond five had no positive effect on the estimation capability of the linear regression. Therefore, a subset of five molecular descriptors was considered, and the determined descriptors are presented in Table 2.

**Table 2:** The selected molecular descriptors as chemical variables.

Molecular descriptor	Descriptor type	Definition
Lop	Topological descriptors	Lopping centric index
CIC2	Information indices	Complementary information content (neighborhood symmetry of 2-order)
EEig12x	Edge adjacency indices	Eigenvalue no. 12 from edge adj. matrix weighted by edge degrees
BEHp2	Burden eigenvalue descriptors	Highest eigenvalue no. 2 of Burden matrix/weighted by atomic polarizabilities
G3s	WHIM descriptors	3rd component symmetry directional WHIM index/weighted by atomic electro-topological states

### Developing and validation of linear multi-variable model for CMC

The determined descriptors along with T, S_eq_ and pH were utilized to generate a multivariate linear regression model for CMC. To evaluate the predictive performance of the model, several common statistical criteria were emplyed. The root-mean-square deviation (RMSD), mean absolute error (MAE), and R^2^ which are widely used parameters, were utilized in this study.

11 $${\text{RMSD = }}\sqrt {\left( {\frac{1}{{\text{n}}}} \right)\sum\limits_{{\text{i = 1}}}^{{\text{n}}} {{\text{(y}}_{{\text{i}}}^{{{\text{exp}}{.}}} - {\text{y}}_{{\text{i}}}^{{{\text{cal}}{.}}} {)}^{{2}} } }$$

12 $${\text{MAE = }}\left( {\frac{1}{{\text{n}}}} \right)\sum\limits_{{\text{i = 1}}}^{{\text{n}}} {\left| {{\text{y}}_{{\text{i}}}^{{{\text{exp}}{.}}} - {\text{y}}_{{\text{i}}}^{{{\text{cal}}{.}}} } \right|}$$

${\text{y}}_{{\text{i}}}^{{{\text{exp}}{.}}}$,${\text{y}}_{{\text{i}}}^{{{\text{cal}}{.}}}$, and n represent the experimental, estimated and number of samples of the dependent variable in the dataset, respectively. Lower values of RMSD and MAE, which indicate proximity to zero, are more desirable. The R^2^ value should be close to unity. In addition to the common statistical criteria, several specific statistical techniques are used in the QSPR modeling approach to validate any QSPR linear model. The main QSPR validation methods include leave-one-out (LOO) cross-validation, leave-N-out (LNO) cross-validation, bootstrapping, y-randomization, and external validation. Although the explanation of these specific techniques has been proposed in some studies^60^,a brief review is presented here.

In LOO cross-validation, each sample in the training dataset is excluded once, and a new multivariate linear regression is generated without that sample. Using the new regression, the dependent variable of the excluded sample is estimated. The values of the correlation coefficient (Q^2^) and root mean square error of cross-validation (RMSECV) are then computed using the following equations:

13 $${\text{RMSECV = }}\sqrt {\left( {\frac{1}{{\text{n}}}} \right)\sum\limits_{{\text{i = 1}}}^{{\text{n}}} {{\text{(y}}_{{\text{i}}}^{{{\text{exp}}{.}}} - {\text{y}}_{{\text{i}}}^{{{\text{cal}}{.}}} {)}^{{2}} } }$$

14 $${\text{Q}}^{{2}} { = 1 } - \, \frac{{\sum\nolimits_{{\text{i = 1}}}^{{\text{n}}} {{\text{(y}}_{{\text{i}}}^{{{\text{exp}}{.}}} - {\text{y}}_{{\text{i}}}^{{{\text{cal}}{.}}} {)}^{{2}} } }}{{\sum\nolimits_{{\text{i = 1}}}^{{\text{n}}} {{\text{(y}}_{{\text{i}}}^{{{\text{exp}}{.}}} - \overline{{\text{y}}}^{\exp .} {)}^{{2}} } }}$$

where ${\text{y}}_{{\text{i}}}^{{{\text{exp}}{.}}}$,${\text{y}}_{{\text{i}}}^{{{\text{cal}}{.}}}$, $\overline{{\text{y}}}^{\exp .}$, and n represent the experimental, estimated, average of experimental values, and the number of samples in the training dataset, respectively.

LNO cross-validation is similar to LOO, with the only difference being that in LNO cross-validation, a group of samples is excluded instead of just one. The values of RMSECV and Q^2^ are recalculated for LNO cross-validation. In LOO cross-validation, repeating the test does not affect RMSECV and Q^2^. However, in LNO ross-validations, RMSECV and Q^2^ can vary due to the repetition of the test. In this study, the LNO cross-validation test was repeated three times and the results were reported. In developing a QSPR linear model, the minimum acceptable values for statistical variables are Q^2^ > 0.5 and R^2^ > 0.6. A difference between Q^2^ and R^2^ that exceeds 0.2–0.3 indicates overfitting in the QSPR linear modeling process^60^.

In the bootstrapping technique, the entire dataset is randomly divided into training and test datasets multiple times. For each split, a respective multivariate linear regression is generated, and LOO cross-validation is performed. The values of R^2^ and Q^2^ are then calculated and their averages are reported (i.e. R^2^_boot_ and Q^2^_boot_). In bootstrapping, a data point may be excluded once, multiple times, or never. In the present study, bootstrapping was performed 5000 times.

The y-randomization method is used to assess the possibility of chance correlation between the dependent and independent variables of a QSPR linear model. In the y-randomization test, the original matrix of independent variables values is fixed, and the vector of dependent variable is randomized. A regression is then constructed between the randomized variables. If there is no chance correlation, the resulting multivariate regression should be of poor quality. Y-randomization is performed multiple times, and the values of R^2^ and LOO correlation coefficient (Q^2^) are calculated for each regression (i.e. R^2^_yi_ and Q^2^_yi_). The results of y-randomization are usually presented graphically as R^2^_i_ versus Q^2^_i_. When Q^2^_yi_ < 0.2 and R^2^_yi_ < 0.2, there is no chance correlation risk^14,60^. In the present study, y-randomization was performed 1000 times.

External validation is another method in which the main dataset is randomly split into structurally similar sets of training data and an external validation set (i.e., a test set). In the present study, at first, 10% of the entire dataset was randomly selected as the external validation set (i.e., the test set) and was used to evaluate the estimation applicability.

After developing and evaluating the multi-variable linear model, the SGB and GP algorithms were applied to generate nonlinear models for CMC using the independent variables (i.e. the determined descriptors, T, and S_eq_). Nonlinear models often provide more accuracy and estimation power.

### Stochastic gradient boosting (SGB)

In the current inquiry, the stochastic gradient boosting (SGB) tree framework was implemented over collected data to model CMC.

Stochastic Gradient Boosting is an improvement on the classic Gradient Boosting method, created by Friedman^61^. By incorporating Breiman's bagging approach^62^, it boosts accuracy and efficiency by randomly sampling the training data^63,64^. This results in better prediction performance^65^, and the technique has been proven effective in many industries and applications^66–76^.

In more general terms, Gradient Boosting (GB) is an effective algorithm that transforms weak hypotheses into strong ones by combining a series of ensemble learners made up of simple base or weak learners^77,78^. A weak learner is defined as one whose performance is only slightly better than random chance, and in the case of GB, decision trees (such as regression trees) are commonly used as weak learners. To avoid overfitting, the construction of trees is often constrained by limiting the number of levels or choosing the best split points based on minimizing a loss function.

The overall goal of the algorithm is to minimize the loss of the model by adding weak learners using a gradient descent-like procedure. At each iteration, a new weak learner is added that focuses on the cases that the previous weak learner did not predict correctly, thus reducing the loss. The output of each generated tree is then added to the output of the sequence of trees to gradually improve the final output of the model.

Stochastic GB is a variation of GB where a subsample of the total training set is randomly selected for each iteration, and the base learner is fit on that subsample without replacement^61,64^. This reduces the risk of overfitting and allows for self-validation of the model internally by using out-of-bag error estimates. Additionally, the algorithm becomes faster since regression trees are generated on smaller datasets at each iteration. The review of the literature has shown the high ability of this new branch of decision tree algorithm in chemical engineering areas^79,80^.

**Figure 2:**
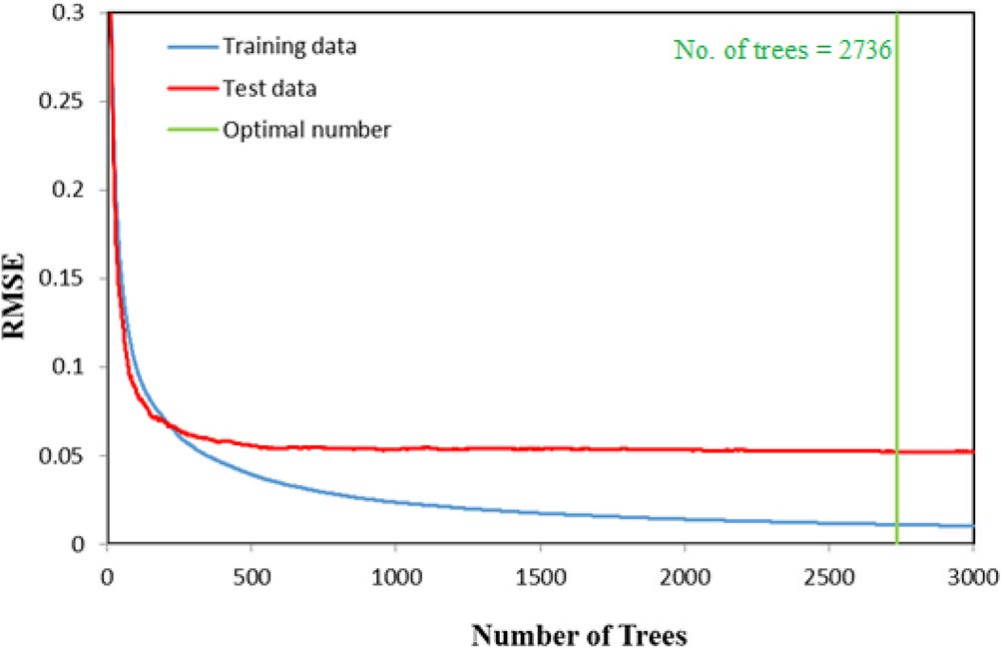
The graph of RMSD over the successive boosting steps for the training and test samples using SGB method.

When developing the SGB model, the error values sharply decreased with an increasing number of trees until the error rate stabilized (see Fig. 2). The SGB algorithm selected a solution with 2736 number of trees, which was the solution that returned the minimum error in the form of RMSD for the test data set (RMSD_test_ = 0.05203).

**Figure 3:**
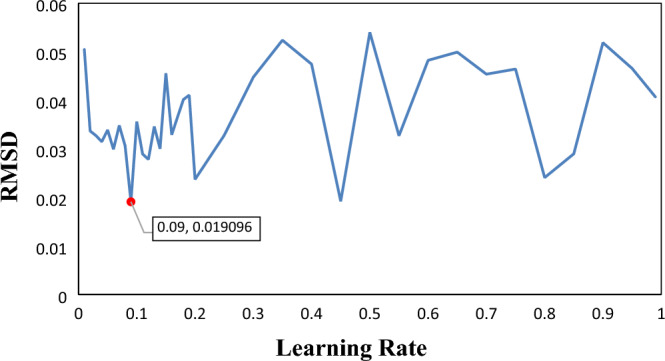
The effects of learning rate on the performance of the SGB model for predicting CMC.

To achieve the most generalizable model, determining the learning rate was crucial. The learning rate is the specific weight at which consecutive simple trees are added to the prediction equation, and it is considered the most important parameter. To identify the optimal value, a sensitivity analysis was performed, which demonstrated the effects of learning rate on the performance of the SGB model for predicting CMC, as illustrated in Fig. 3. The optimized parameter was determined to be 0.09. Using the SGB tree, the importance degrees of all the model parameters were also determined.

### Genetic programming (GP)

Genetic programing (GP) is an algorithm used in the present study to develop the CMC model. GP is a well-known machine learning approaches for optimization and modeling studies which was introduced in the 1990s by John Koza^81^. The GP procedure is inspired by biological generation phenomenon in which computer programs evolve evolutionarily in a machine learning algorithm to perform tasks.

**Figure 4:**
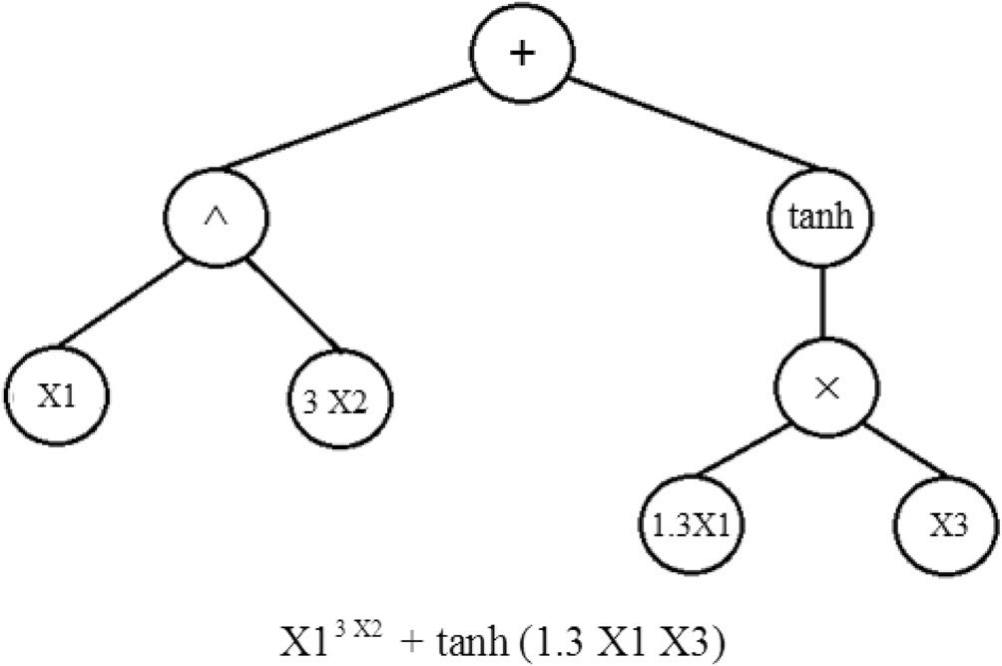
Schematic of a simple GP gene including the operators: + , ^, × , tanh.

In the GP process, a population of mathematical functions is first randomly generated from pre-determined user-defined mathematical operators. Then, some of these functions are randomly chosen to be arranged in the form of one or several “genes”. A Gene is represented as a chromosome-like syntactic tree structure that operates on input data, i.e., the training dataset(as shown in Fig. 4)^82,83^.

After the primary genes are determined from the first population (known as parents), the overall primary GP model is developed by a weighted summation of the genes with a bias term. However, the primary model does not provide the desired accuracy, and a modification process is required. In the next step, the tree structures of primary genes are modified by crossing over the best performing trees and cutting some sections of trees to be exchanged between themselves. This modification mainly results in a new population (next generation or children) due to changes in the mathematical functions^84^.

The generation is iterated several times in a regular process until the last population is generated, which includes the most-optimized functions with a specific arrangement of genes to solve the problem^85^. In the modeling applications of GP, regression between the objective function and independent variables is also known as “multi-gene symbolic regression”. It is an effective technique that includes one or more genes (individual usual GP trees) providing simple and fast processing to perform tasks^83,86^.

In this study, the number of populations and number of generations were set as 180 each, and the mathematical operators + , −, × , /, and exp (exponential) were employed. GP was run over the input data, and the output model with acceptable accuracy was obtained.

## Results and discussion

### Multi-variable linear correlation of CMC

The multi-variable linear model for CMC of anionic surfactants in brine is presented below:

15 $$\begin{aligned} {\text{Log}}_{10} \left( {CMC} \right) & \, = \, 31.817705( \pm 1.59767) \, + \, 0.002290( \pm 0.00066) \, \times {\text{ T}} - 0.083577( \pm 0.02999) \, \times {\text{ pH}} \\ \, & \quad - 0.000023( \pm 0.000002) \, \times {\text{ S}}_{{{\text{eq}}}} - 0.498878( \pm 0.03992) \times \{ {\text{CIC}}2\} - 0.465377( \pm 0.03149) \times \{ {\text{EEig}}12{\text{x}}\} \\ \, & \, \quad - \, 0.445544( \pm 0.05699) \times \{ {\text{Lop}}\} - 7.805830( \pm 0.44219) \times \{ {\text{BEHp}}2\} - 2.840368( \pm 0.36536) \times \{ {\text{G}}3{\text{s}}\} \\ \end{aligned}$$

The variables of the new developed model have been presented in Tables 1 and 2. The determined descriptors (shown in Table 2) are “CIC2”^87^, “EEig12x”^88^, “Lop”^88,89^, “BEHp2”^90^, and “G3s”^91^.

CIC2 is a complementary information content of 2nd order neighborhood symmetry from the category of information indices descriptors. It is a measure of the degree of diversity of elements in the structure^87^.

The Lop descriptor is a lopping centric index categorized in topological descriptors, which are usually obtained from a hydrogen-depleted molecular graph. A molecular graph is a labeled graph whose vertices correspond to the atoms of the compound labeled with the kinds of atoms, and the edges correspond to chemical bonds labeled with the types of bonds^89^.

Lop is an index defined as the mean information content derived from the pruning partition of a graph^88^.

EEig12x is one of the edge adjacency indices descriptors, which stands for the 12th eigenvalue of the edge adjacency matrix weighted by edge degrees. The edge adjacency matrix derived from a molecular graph encodes the connectivity between graph edges^88^.

BEHp2 belongs to the Burden eigenvalue category from 2D topological descriptors. It is a measure of molecule/ion polarizability defined as the 2nd highest eigenvalue of the Burden matrix, which is weighted by atomic polarizabilities^90,92^.

G3s is a WHIM descriptor and is defined as the 3rd component symmetry directional WHIM index weighted by atomic electro-topological states. WHIM specifications are used to calculate 3D molecular information based on molecular size, shape, symmetry, diversity of atoms, etc.^91^.

**Figure 5:**
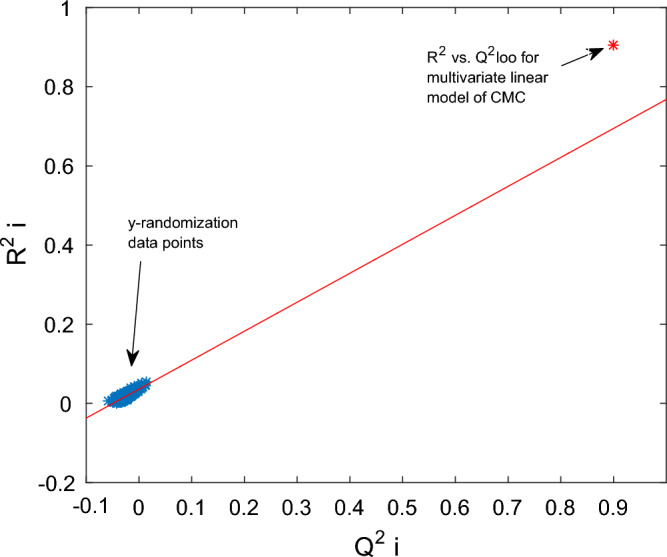
The result of y-randomization test for multi-variable linear model of CMC.

The statistical parameters of the multivariate linear correlation, including QSPR specific validation parameters, are presented in Tables 3 and 4. The values of R^2^, RMSD, and MAE show medium accuracy of the linear model. The validity of the linear model was checked by LOO cross-validation, LNO cross-validation, bootstrapping, y-randomization, and external validation techniques. The LNO cross-validation parameters are shown in Table 4, and the bootstrapping test was performed 5000 times. The low difference between the values of Q^2^_LOO_, Q^2^_LNO_, Q^2^_boot,_ Q^2^_ext_, R^2^_boot_, and R^2^_ext_ indicates that the linear model has been developed without occurring overfitting. The y-randomization test was repeated 1000 times, and the results are shown in Fig. 5. According to this test, the values of Q^2^_yi_ and R^2^_yi_ (i.e., y-randomization data points) are of poor quality compared to the linear model correlation coefficient (R^2^) and Q^2^_LOO_ (indicated as a red point in Fig. 5), which verifies that there is no risk of chance correlation in the multi-variable linear model of CMC.

**Table 3:** Statistical parameters of multivariate linear model for CMC of anionic surfactants in brine.

n _total_ = 488	n _train_ = 440	n _test_ = 48
R^2^_total_ = 0.9059	R^2^_train_ = 0.9061	R^2^_test_ = 0.9047
RMSD _total_ = 0.2382	RMSD _train_ = 0.2367	RMSD _test_ = 0.2514
MAE _total_ = 0.1734	MAE _train_ = 0.1711	MAE _test_ = 0.1947
Q^2^_LOO_ = 0.8988	RMSECV _LOO_ = 0.2456	Q^2^_boot_ = 0.8990
R^2^_boot_ = 0.9064	Q^2^_ext_ = 0.9048	R^2^_ext_ = 0.9047

The subscripts “total”, “train” and “test” are attributed to total dataset, training dataset and test dataset, respectively.

**Table 4:** Statistical parameters of LNO cross-validation for linear model of CMC.

1st	2nd	3th	Average
Q^2^_L – 25% – O_ = 0.8940	Q^2^_L – 25% – O_ = 0.8943	Q^2^_L – 25% – O_ = 0.8991	Q^2^_L – 25% – O_ = 0.8958
RMSECV _LNO_ = 0.2514	RMSECV _LNO_ = 0.2511	RMSECV _LNO_ = 0.2453	RMSECV _LNO_ = 0.2493

**Figure 6:**
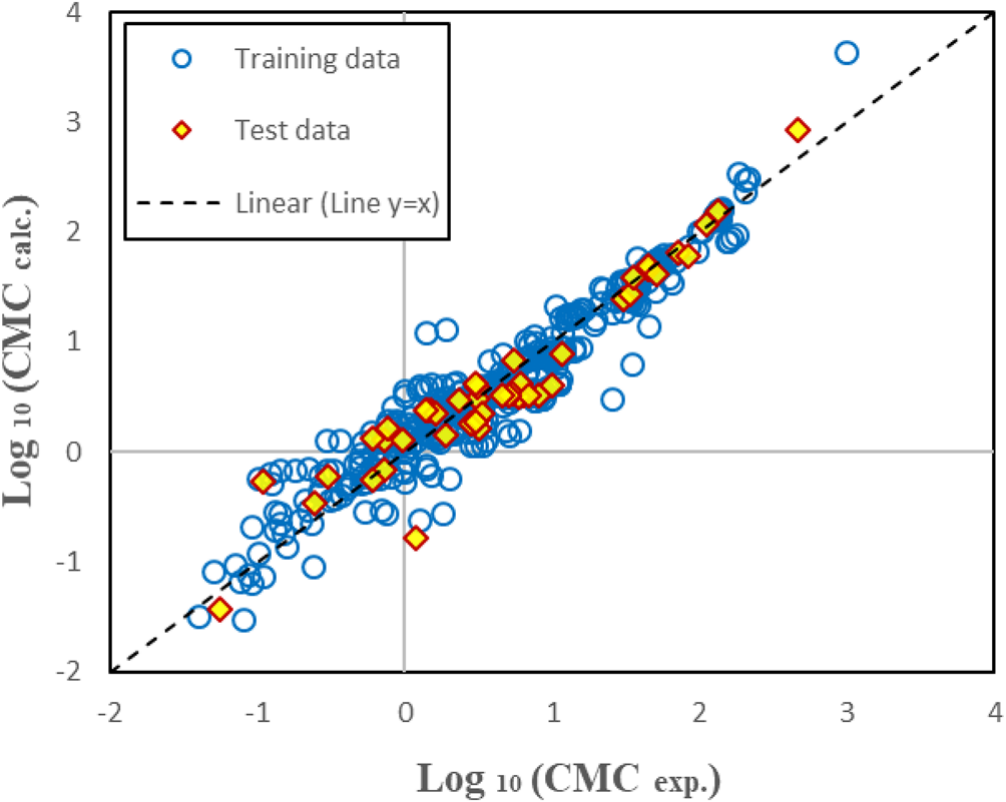
The estimated CMC versus experimental data for multivariate linear model over training and test datasets.

The estimated CMC by Eq. (15) versus experimental data is presented in Fig. 6. Based on Tables 3 and 4 and Fig. 6, the linear model has acceptable accuracy. However, the prediction ability is not excellent enough. The results of non-linear models are proposed in the next section.

### Non-linear models of CMC

The SGB and GP programs were run over the input data to produce new models for the CMC of anionic surfactants in a brine solution. The execution of the SGB algorithm in this study follows the explanations in Friedman^61,64^. The new GP model is a mathematical relation as follows:

16 $$\begin{aligned} {\text{Log}}_{10} \left( {{\text{CMC}}} \right) \, & { = }0.0006095{\text{ S}}_{{{\text{eq}}}} - {13}{\text{.76}}\left\{ {{\text{CIC2}}} \right\}{ + 0}{\text{.0003308}}\left\{ {{\text{EEig12x}}} \right\} - {6}{\text{.882}}\left\{ {{\text{BEHp2}}} \right\}{ + 0}{\text{.001219}}\left\{ {{\text{EEig12x}}} \right\}^{{2}} \\ & \quad { - 1}{\text{.096 }}\left( {{\text{exp}}\left( {{ - }\left\{ {{\text{EEig12x}}} \right\}\left( {\left\{ {{\text{EEig12x}}} \right\}{ + }\left\{ {{\text{G3s}}} \right\}} \right)} \right){\text{ + exp}}\left( { - {2}\left\{ {{\text{EEig12x}}} \right\}^{{2}} } \right)} \right){ + 13}{\text{.56 exp}}\left( {{\text{ - exp}}\left( {\left\{ {{\text{G3s}}} \right\} - \left\{ {{\text{CIC2}}} \right\}} \right)} \right) \\ \, & \quad { - }\left( {\left\{ {{\text{BEHp2}}} \right\}{ + }\left\{ {{\text{G3s}}} \right\}} \right)\left( {{0}{\text{.0001654 S}}_{{{\text{eq}}}} + {31}{\text{.68}}\left( {\frac{{\left\{ {{\text{CIC2}}} \right\}{ + }\left\{ {{\text{EEig12x}}} \right\}}}{{{1}{\text{.407 T + 2}}{\text{.815 S}}_{{{\text{eq}}}} }}} \right)} \right){ 0}{\text{.01423}}\left\{ {{\text{EEig12x}}} \right\}\left( {\frac{{{\text{pH + }}\left\{ {{\text{G3s}}} \right\}}}{{\left\{ {{\text{G3s}}} \right\}}}} \right) \\ & \quad { - 3}{\text{.961}}\left( {\left\{ {{\text{CIC2}}} \right\}{ + }\left\{ {{\text{EEig12x}}} \right\}} \right)\left( {\frac{{{\text{S}}_{{{\text{eq}}}} - {9}{\text{.438}}}}{{{9}{\text{.007 (T + S}}_{{{\text{eq}}}} {)}}}} \right){ + 0}{\text{.8278}}\left( {\left\{ {{\text{CIC2}}} \right\}{\text{ - exp}}\left( { - \left\{ {{\text{CIC2}}} \right\}} \right)} \right)\left( {\left\{ {{\text{CIC2}}} \right\}\left( {{1} - \frac{1}{{{\text{pH}}}}} \right){ + 9}{\text{.447}}} \right) \\ & \quad { - 0}{\text{.5913}}\left( {{2}{\text{.908}}\left\{ {{\text{Lop}}} \right\} - \left\{ {{\text{CIC2}}} \right\}} \right){\text{ exp}}\left( {\left\{ {{\text{BEHp2}}} \right\}{ - 5}{\text{.068}}} \right) - { + 0}{\text{.4997 exp}}\left( {{2}\left\{ {{\text{EEig12x}}} \right\} - \left\{ {{\text{CIC2}}} \right\}} \right){ + 26}{\text{.1 }} \\ \end{aligned}$$

**Figure 7:**
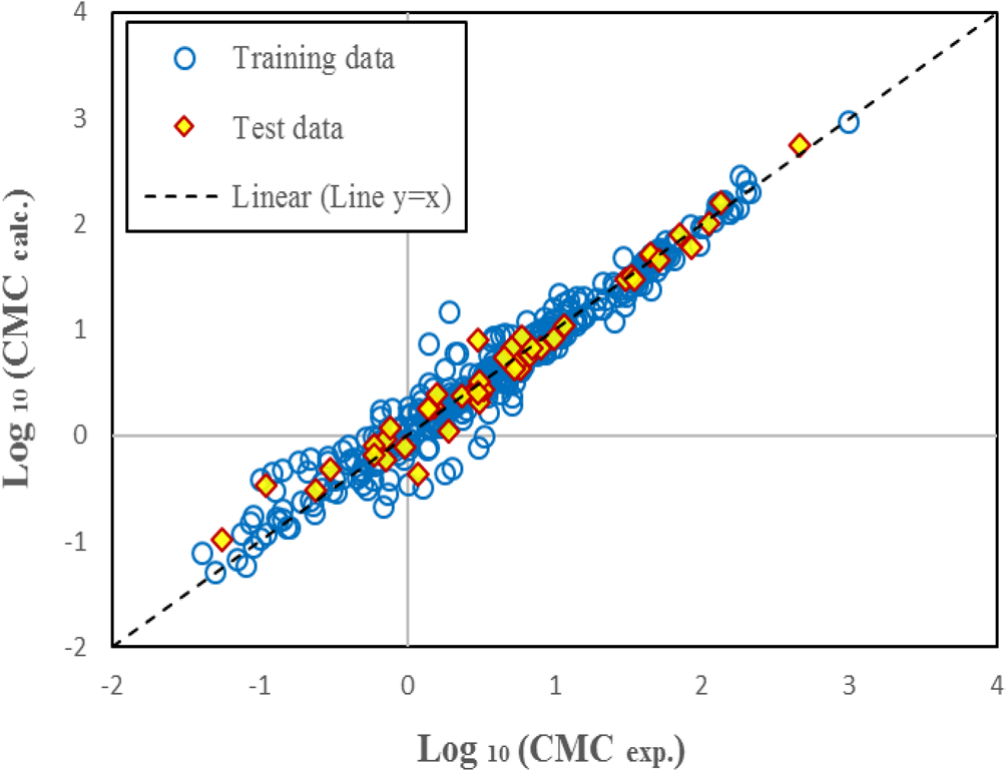
The estimated CMC versus experimental values for GP model over training and test datasets.

**Figure 8:**
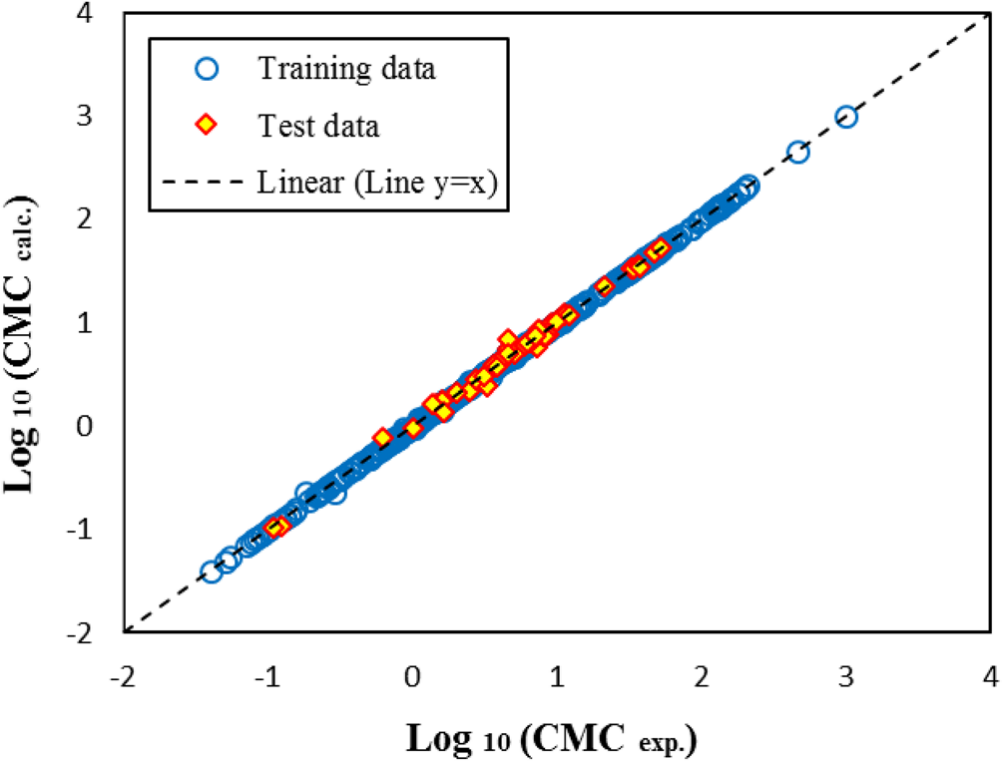
The estimated CMC versus experimental values for SGB model over training and test datasets.

Table 5 shows the statistical parameters of the presented models. The values of R^2^, RMSD, and MAE represent the acceptable applicability of SGB and GP models and the high accuracy and superiority of the SGB method. Figures 7 and 8 show the estimated CMC versus the experimental values for the GP and SGB models, respectively. The calculated data by the SGB model has been scattered well on the 45 degree line (y = x), verifying excellent accuracy.

**Table 5:** Statistical parameters of non-linear models for CMC of anionic surfactants in brine.

Statistical parameters	SGB model			GP model		
	All	Train	Test	All	Train	Test
R^2^	0.999395	0.999808	0.991658	0.954946	0.953866	0.963834
RMSD	0.019096	0.010993	0.052034	0.164829	0.165879	0.154869
MAE	0.008387	0.005457	0.036536	0.111650	0.111228	0.115514

**Figure 9:**
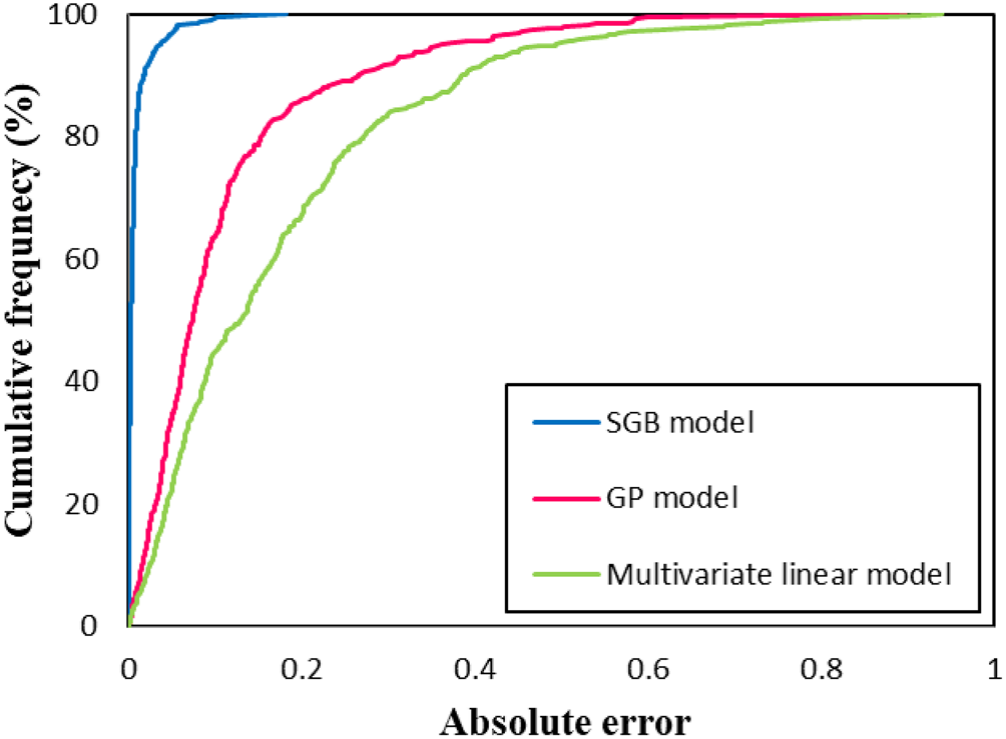
Cumulative frequency of the new developed models.

**Figure 10:**
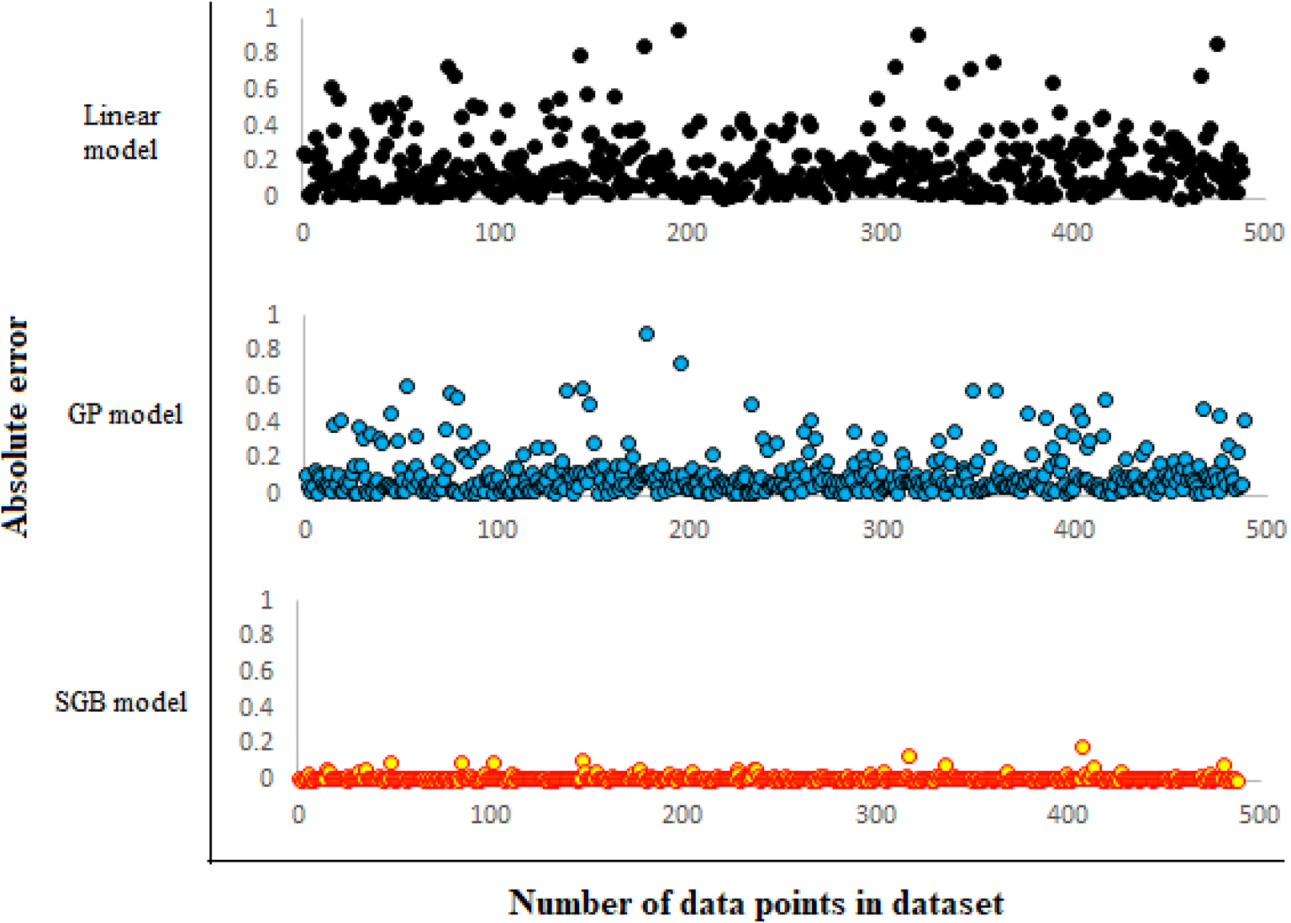
Absolute errors of data points over all dataset for linear model (top), GP model (middle) and SGB model (down). It is observed that the estimation accuracy has been increased from top to down.

Figure 9presents the curves of cumulative frequency versus absolute errors of the objective function (Log _10_ (CMC)) for the SGB and GP models, as well as the linear correlation. The maximum absolute error of the SGB model in this figure is 0.18. Moreover, the absolute errors of 82.2% of all datasets are less than 0.01, and the absolute errors of 99.2% of the data are below 0.1 for the new SGB model. Figure 10 shows absolute errors over the total dataset for the linear (top plot), GP (middle plot), and SGB (bottom plot) models. As observed in Figs. 9 and 10, the estimation accuracy has been enhanced from the linear model to the SGB model, and the accuracy of the SGB method is the highest.

**Figure 11:**
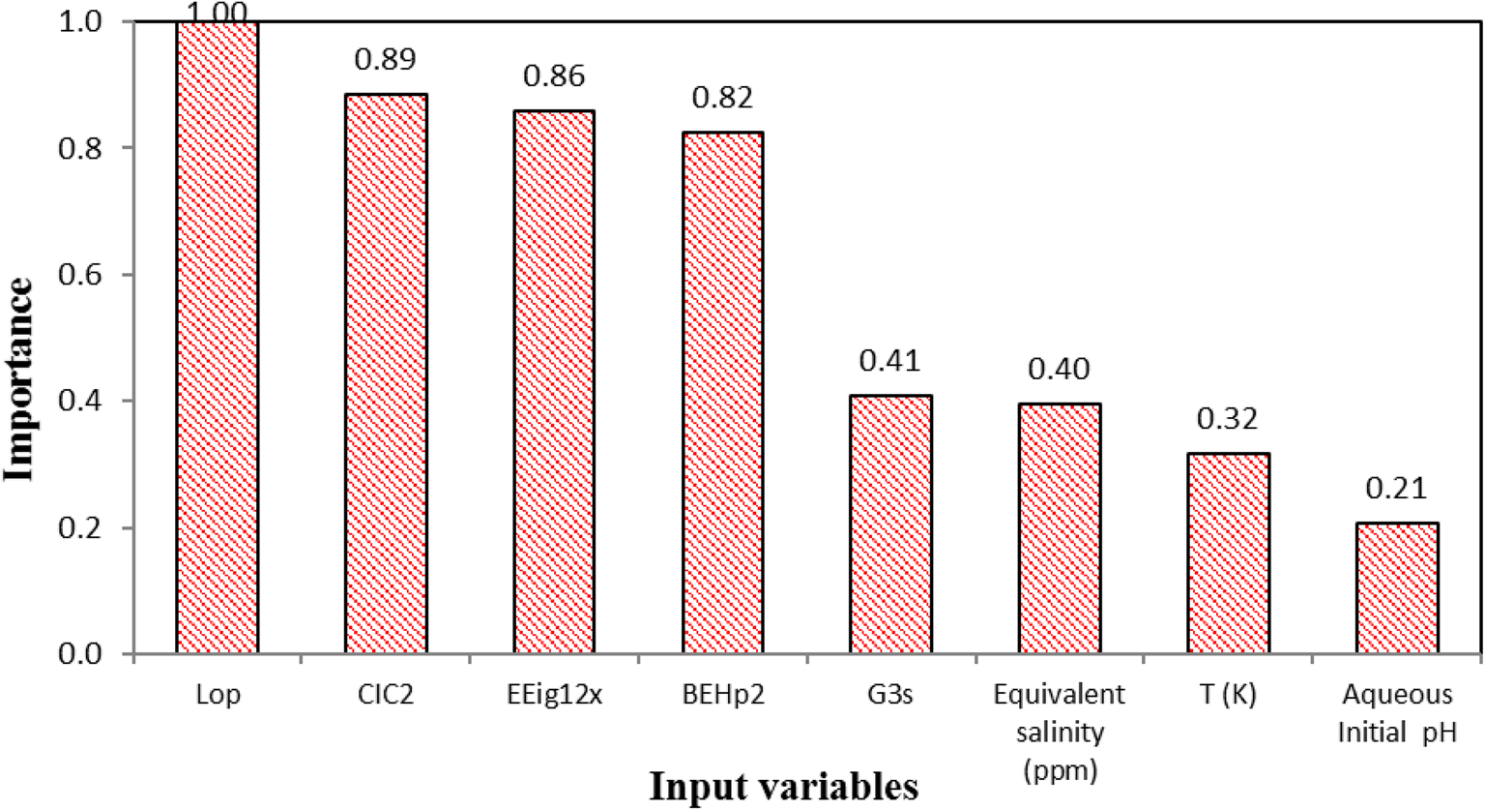
Relative importance of independent variables on the CMC based on the SGB algorithm.

The relative importance of independent variables, including descriptors (Lop, CIC2, EEig12x, BEHp2, and G3s), T, pH, and S_eq_, has been determined by the SGB algorithm in the calibration of the SGB model, and the results have been depicted in Fig. 11. A higher value of a variable indicates stronger relative importance on the response. As shown, the descriptor Lop is the more effective factor among the input variables in the development of the SGB model.

The application of the proposed models has been shown in Table 6 for the estimation of the CMC of sodium dodecyl sulfate as a sample in the dataset.

**Table 6:** Application of the new QSPR models of the present study for estimation of CMC of a sample component.

Surfactant	Structure	Physical variables	Descriptors of anionic part (without Na^+^ ion)	CMC
sodium dodecyl sulfate		T = 298.15 K pH = 7 S_eq_ = 309.75 ppm	Lop = 2.911 CIC2 = 2.838 EEig12x = 0 BEHp2 = 3.574 G3s = 0.275	log (CMC)_exp._ = 0.805 log (CMC)_calc._ _linear model = 0.516 (AE^a^ = 0.289) log (CMC)_calc._ _GP model = 0.729 (AE = 0.076) log (CMC)_calc._ _SGB model = 0.804 (AE = 0.001)

^a^The abbreviation AE indicates absolute error calculated as : AE =|y^exp.^ – y^calc.^|.

The generation of new models with high accuracy for the CMC of surfactant solutions containing different types of salts based on the QSPR approach and the application of GP and SGB for producing non-linear models are novelties of the present study. Using a wide range of salinities and temperatures, as well as various types of anionic surfactants in the modelling procedure, has increased the estimation applicability and prediction performance of the newly developed models.

## Conclusion

The estimation of CMC is one of the most important interests of the academic and industrial communities dealing with surfactants. The present study was conducted to obtain novel methods for the estimation of the CMC of well-known, highly-used anionic surfactants as functions of both physical parameters (T, pH and salinity) and chemical factors (Lop, CIC2, EEig12x, BEHp2, and G3s) and to avoid the expensive and time-consuming laboratory measurements. CMC estimation at different temperatures and salinities is considered novel and innovative. The QSPR molecular approach, along with the ensemble learning framework of stochastic gradient boosting (SGB) and genetic programming (GP) procedures, was used to produce models for CMC in brine. The implemented algorithms are reliable and applicable for predicting CMC. However, the output of SGB is more accurate in terms of statistical parameters. This inquiry also encourages the scientific and engineer communities to further investigate the use of the novel branch of soft computing frameworks. Developing such models for CMC provides new applications in the simulation and control of surfactant systems, as well as prediction of CMC for newly designed anionic surfactants.

## Data Availability

All the literature datasets analyzed in this study are available at a reasonable request from the corresponding authors.

## Abbreviations

AE: Absolute error
ANFIS: Adaptive neuro-fuzzy inference system
ANNs: Artificial neural networks
BEHp2: Highest eigenvalue no. 2 of Burden matrix/weighted by atomic polarizabilities
CEOR: Chemical enhanced oil recovery
CIC2: Complementary information content (neighborhood symmetry of 2-order)
CMC: Critical micelle concentration
D: Dipole moment
EEig12x: Eigenvalue 12 from edge adj. matrix weighted by edge degrees
E_HOMO_: Energy of the highest occupied molecular orbital
E_LUMO_: Energy of the lowest unoccupied molecular orbital
EOR: Enhanced oil recovery
ERM: Enhanced replacement method
E_t_: Total energy of molecule
f-I_BAL_: Balaban distance connectivity index
FSR: Forward stepwise regression
G3s: 3Rd component symmetry directional WHIM index/weighted by atomic electro-topological states
GA-MLR: Genetic algorithm multivariate linear regression
GB: Gradient boosting
GFA: Genetic function approximation
GP: Genetic programming
HGP: Hydrophobic group position
ICA: Imperialist competitive algorithm
KH0: Kier and Hall molecular connectivity index of zero-th order
KH1: Kier and Hall molecular connectivity index of first-order
KS3: Kier shape index of third-order
Lop: Lopping centric index
MAE: Mean absolute error
MM2: Molecular mechanics
*n*: Number of samples in the dataset
N_T_: Total atom number
P: Pressure
PSO: Particle swarm optimization
Q^2^_boot_: LOO cross-validation squared correlation coefficient of bootstrapping
Q^2^_ext_: Squared correlation coefficient of external-validation
Q^2^_LNO_: Leave-N-out cross-validation squared correlation coefficient
Q^2^_LOO_: Leave-one-out cross-validation squared correlation coefficient
Q^2^_yi_: Y-randomization LOO cross-validation squared correlation coefficient
Q_C-max_: Maximum net atomic charges on carbon atom
QSPR: Quantitative-structural property relationship
R^2^: Squared correlation coefficient
R^2^_boot_: Squared correlation coefficient of bootstrapping test
R^2^_ext_: Squared correlation coefficient of external-validation test
R^2^_yi_: Squared correlation coefficient of y-randomization test
RA^−1^: Reciprocal of randic index
RM: Replacement method
RMSD: Root-mean-square deviation
RMSECV: Root-mean-square error of cross-validation
RSD: Residual standard deviation
RNC: Relative number of carbon atoms
S_eq_: NaCl equivalent salinity
SGB: Stochastic gradient boosting
SVM: Support vector machine
T: Temperature
TDIP: Total dipole moment
WHIM: Weighted holistic invariant molecular
WI: Wiener number
${\text{y}}_{{\text{i}}}^{{{\text{cal}}{.}}}$: Predicted dependent variable
${\text{y}}_{{\text{i}}}^{{{\text{exp}}{.}}}$: Experimental dependent variable
$\overline{{\text{y}}}^{\exp .}$: Average of experimental dependent variable
ΔH_f_: Molar heat of formation
Π: Octanol/water partition coefficient
